# Immunoregulatory Effects of Porcine Plasma Protein Concentrates on Rat Intestinal Epithelial Cells and Splenocytes

**DOI:** 10.3390/ani11030807

**Published:** 2021-03-13

**Authors:** Cristina Hernández-Chirlaque, Carlos J. Aranda, Borja Ocón, Javier Polo, Olga Martínez-Augustin, Fermín Sánchez de Medina

**Affiliations:** 1 Department of Biochemistry and Molecular Biology II, School of Pharmacy, CIBERehd, Instituto de Investigación Biosanitaria ibs.GRANADA, University of Granada, 18071 Granada, Spain; cristinahech@hotmail.com (C.H.-C.); cjarandaclemente@gmail.com (C.J.A.); 2 Department of Pharmacology, CIBERehd, School of Pharmacy, Instituto de Investigación Biosanitaria ibs.GRANADA, University of Granada, 18071 Granada, Spain; borjaoconel@gmail.com (B.O.); fsanchez@ugr.es (F.S.d.M.); 3 APC Europe S.L.U., 08403 Granollers, Spain; javier.polo@apc-europe.com

**Keywords:** immunoglobulin concentrate, serum protein concentrate, Toll-like receptors, TLR4, Myd88

## Abstract

**Simple Summary:**

Blood contains proteins which have interest as products that may regulate immune function. For this reason some protein-based products are currently used as nutritional supplements for animals, for instance two porcine concentrates, spray dried serum (SDS), and an immunoglobulin concentrate (IC). These products have shown to protect against colonic inflammation in rodents. In the present study we characterize the ability of these products to modulate immune function in isolated cells, namely intestinal epithelial cells (IEC18 cells) and rat spleen cells. Our data indicate that both porcine protein concentrates indeed alter immune cell function, based on the secretion of the modulators known as cytokines. In intestinal epithelial IEC18 cells they promoted the secretion of GROα and MCP-1 cytokines. In spleen cells they mainly inhibited the production of TNF, a key proinflammatory cytokine. In addition, the IC product augmented the release of IL-10, an anti-inflammatory cytokine. Taken together, our data indicate that the immunomodulatory effects observed in vivo are consistent with the direct actions of the protein concentrates on epithelial cells, T lymphocytes, and monocytes.

**Abstract:**

Serum protein concentrates have been shown to exert in vivo anti-inflammatory effects. Specific effects on different cell types and their mechanism of action remain unraveled. We aimed to characterize the immunomodulatory effect of two porcine plasma protein concentrates, spray dried serum (SDS) and an immunoglobulin concentrate (IC), currently used as animal nutritional supplements with established in vivo immunomodulatory properties. Cytokine production by the intestinal epithelial cell line IEC18 and by primary cultures of rat splenocytes was studied. The molecular pathways involved were explored with specific inhibitors and gene knockdown. Our results indicate that both products induced GROα and MCP-1 production in IEC18 cells by a MyD88/NF-κB-dependent mechanism. Inhibition of TNF production was observed in rat primary splenocyte cultures. The immunoglobulin concentrate induced IL-10 expression in primary splenocytes and lymphocytes. The effect on TNF was independent of IL-10 production or the stimulation of NF-kB, MAPKs, AKT, or RAGE. In conclusion, SDS and IC directly regulate intestinal and systemic immune response in murine intestinal epithelial cells and in T lymphocytes and monocytes.

## 1. Introduction

Protein products have interest for immunomodulation and the promotion of health beyond their nutritional properties, i.e., as functional foods or nutritional supplements. Thus, spray dried plasma (SDP) has been used as a dietary supplement to promote animal growth. This approach has also been reported to exert protection against rotavirus diarrhea in pigs [1]. Several of these concentrates from bovine or porcine origin have been shown to affect the host response in animal models of intestinal and lung inflammation [2,3,4,5,6,7,8]. Namely, in young and senescent animals challenged by *Staphylococcus aureus* enterotoxin B, dietary supplementation with functional proteins from SDP of porcine origin has been shown to modulate the intestinal barrier and defense mechanisms, thereby reducing the degree of gut-associated lymphoid tissue activation [2,8,9]. A dietary supplement containing over 90% bovine serum protein (>50% immunoglobulins) reduces inflammatory markers and tissue damage in mice models of colitis [10,11,12] and mucositis [13]. Simultaneous protection against lung and colon inflammation has been documented for porcine SDP [14]. Further, the latter has been recently shown to exert neuroprotective activity in a senescence murine model [15] and partial protection against intraperitoneal LPS challenge in pregnant mice [16]. On the other hand, immunoglobulin concentrates from porcine or ovine blood display neutralizing activity in vitro against lipopolysaccharide (LPS) and pathogenic Gram-positive and Gram-negative bacteria [17,18]. They have been also used to transfer passive immunity as components of colustrum supplements or replacers [19].

Besides animal studies, clinical trials have shown benefit of immunoglobulin concentrate (IC) administration to patients with irritable bowel syndrome, reducing symptoms (improved stool consistency and frequency, pain, and bloating) and cytokine production [20]. Immunoglobulin concentrates have been also shown to be effective in the management of enteropathy associated with diarrhea-predominant in irritable bowel syndrome and human immunodeficiency virus infection [21,22], or diarrhea produced by *E. coli* or *Shigella* [23,24]. Supplementation of infant formula with an IC protects against diarrhea in infants [25]. The antibodies contained in these products resist digestion in humans and retain bioactivity [26], as shown for a *Clostridium difficile* IC [27,28]. These studies have shown that IC may act by binding antigens in the intestinal lumen [29].

Despite the available in vivo evidence there are a few mechanistic studies dealing with the actions of these protein products on cells. Whey proteins have been shown to stimulate the production of proinflammatory cytokines (IL-6 and IL-8) by the intestinal epithelial cell line Caco-2 [30]. Our study aims to describe the molecular mechanisms involved in the modulation of the immune system by two plasma concentrates from porcine origin, namely spray-dried serum (SDS) and IC. The intestinal cell line IEC18, spleen cells and primary cultures of spleen T lymphocytes and monocytes were used. Signal transduction pathways involved have also been investigated.

## 2. Materials and Methods

### 2.1. Animals

Thirty-two female Wistar rats (190–220 g) obtained from Janvier Labs (Le Gen-est-Saint-Isle, France) were housed in makrolon cages, maintained with a 12 h light–dark cycle and fed standard rodent chow (Panlab A04, Panlab, Barcelona, Spain) and water ad libitum throughout the experiment. All animal experiments were carried out in strict accordance with the ARRIVE guidelines and the EU Directive 2010/63/EU for animal experiments, and were approved by the local ethical committee (ref. 01/03/2017/029).

### 2.2. Materials

Except indicated otherwise, all chemicals were obtained from Sigma-Aldrich (Madrid, Spain). Protein concentrates were supplied by APC Europe, S.L.U. (Granollers, Spain). SDS was obtained after fibrinogen precipitation in acid conditions, then the resulting serum was neutralized to pH 6. IC was obtained from porcine serum following the method described by Lee et al. [31] using sodium hexametaphosphate to obtain an immunoglobulin enriched fraction that was neutralized to pH 7. Both products were ultrafiltered and diafiltered through a 30 kD membrane and the retentate was dried in a Buchi 190 Mini Spray Drier (Büchi Labortechnick AG, Flawil, Switzerland).

### 2.3. Primary Splenocytes

Primary splenocytes were obtained from rats sacrificed by cervical dislocation by mechanical dissection as described before [32] and cultured at 10^6^ cells/mL with or without lipopolysaccharide (LPS, *E. coli* 055:B5, 1 µg/mL). The culture medium was Roswell Park Memorial Institute (RPMI) supplemented with 100 mg/L streptomycin, 100,000 U/L penicillin, 2.5 mg/L amphotericin B and 50 µM β-mercaptoethanol. The cells were maintained at 37 °C in standard culture conditions.

### 2.4. IEC18 Experiments

Non-transformed rat small intestinal epithelial IEC18 cells (ECACC 88011801) (passages 25–50) were obtained from the Cell Culture Service of the University of Granada and cultured in Dulbecco’s modified Eagle’s medium (DMEM) containing fetal bovine serum (10%) until confluency (4–5 days after seeding). All experiments were carried out with DMEM containing no fetal bovine serum, except for some groups as a reference. The control group received PBS. Concentration response curves were initially obtained with cells incubated for 24 h with the protein concentrates. MCP-1 and GROα secretion was measured by ELISA in cell media (Becton Dickinson, Madrid, Spain, and R&D Systems, Minneapolis, MN, sensitivity 10 and 1.3 pg/mL).

In order to explore downstream signaling pathways, IEC18 confluent monolayers were exposed to Bay 11-7082 (10 µM), a selective inhibitor of IκB-α phosphorylation that blocks the NF-κB signaling pathway, or wortmannin (1 µM), an inhibitor of phosphatidylinositol 3 kinase (PI3K) phosphorylation that inhibits the AKT signaling pathway. MAPK inhibitors SB203580 (for p38, 10 µM), PD98059 (for ERK1/2, 10 µM), or SP600125 (for JNK, 10 µM) were also added to the cell cultures to selectively inhibit their phosphorylation. All the inhibitors were dissolved in dimethyl sulfoxide (DMSO) and were added to the culture medium 2 h before the treatment with SDS or IC. DMSO was also added to the control group.

### 2.5. Gene Silencing

In some experiments IEC18 with MyD88 or TLR4 siRNA gene knockdown were used in order to assess the involvement of these signaling molecules in the effects of the products as previously described [33]. In all cases the cell culture medium was free of fetal bovine serum.

### 2.6. Splenocyte Experiments

Rat splenocytes were cultured at 10^5^ cells/100 µL and treated with SDS/IC in basal conditions or under exposure to LPS. The control group received PBS. Cell culture medium was collected after 24 h, cleared by centrifugation, and frozen at −80 °C until assayed for cytokine content by commercial ELISAs (Beckton Dickinson, Madrid, Spain). In all the experiments, samples were run in triplicate and results are expressed as cytokine concentration (pg/mL). Hence *n* = 6 per condition since the experiments were performed twice.

In some experiments NF-κB and MAPK inhibitors were applied as above and the inhibitor of the receptor for advanced glycation end products (RAGE), FPS-ZM1-1, was also used. The involvement of the CD40/IL-10 pathway was assessed with an IRAK1/4 inhibitor (1-(2-(4-Morpholinyl)ethyl)-2-(3-nitrobenzoylamino)benzimidazole) and blocking monoclonal antibodies (anti-CD154 and anti-IL-10, Thermo Fisher Scientific, Waltham, MA, USA), which were added to cells 2 h before other treatments.

### 2.7. Isolation of Splenic T Lymphocytes and Monocytes

Primary T lymphocytes and monocytes were obtained from rat spleen by negative magnetic separation using Miltenyi Biotec microbeads (Madrid, Spain) and columns and BD Biosciences monoclonal antibodies (anti-CD11b, anti-CD161, anti-CD45RA and anti-CD3, anti-CD161, anti-CD45RA, respectively) [32]. Separation and purification protocols were set up and validated by flow cytometry, using FACS Calibur^TM^ (BD Biosciences, San Jose, CA, USA). T cell purity was typically >95%. Cells were cultured as described for splenocytes above.

### 2.8. Statistical Analysis

**Figure 1:**
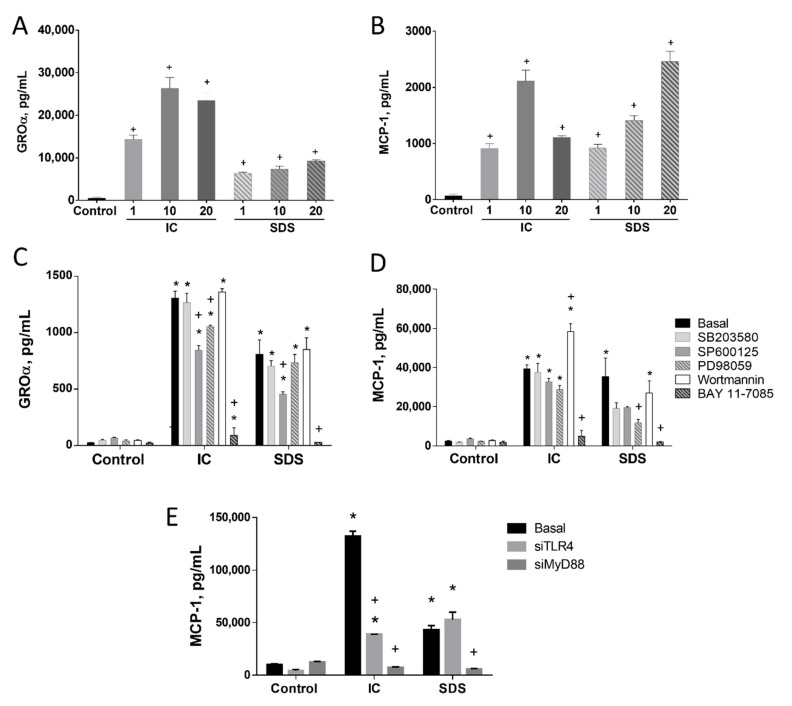
Effect of IC and SDS on cytokine production by IEC18 intestinal epithelial cells. Confluent monolayers were incubated for 24 h with immunoglobulin concentrate (IC) or spray-dried serum (SDS) at 1, 10, and 20 mg/mL and growth-related oncogene α (GROα, (**A**)) or monocyte chemoattractant protein (MCP-1, (**B**)) were measured in the supernatant by ELISA. To assess the mechanism inhibitors for p38 MAPK (SB203580, 10 µM), JNK (SP600125, 10 µM), ERK1/2 (PD98059, 10 µM), phosphatidylinositol 3-kinase (wortmannin, 1 μM) or NF-κB (Bay 11–7085, 10 μM) were added to confluent IEC18 intestinal epithelial monolayers 2 h before the addition of IC or SDS (10 mg/mL) and GROα (**C**) and MCP-1 (**D**) were measured in the 48 h supernatant by ELISA. (**E**) Effect of MyD88 and TLR4 gene silencing on IC and SDS-induced MCP-1 secretion in IEC18. Cells were cultured with IC and SDS (both, 10 mg/mL) for 24 h and MCP-1 was measured in the culture medium by ELISA. Data are represented as mean ± SEM and are representative of two independent experiments (*n* = 3–4). * *p* < 0.05 vs. the corresponding Control group; ^+^ *p* < 0.05 vs. the corresponding Basal (no inhibitor or control vector) group. Results are represented as mean ± SEM and are representative of two independent experiments (*n* = 3).

The data shown are expressed as mean ± SEM and are representative of 2–3 experiments, except those displayed in Figures 1C,D and 6. Means were tested for significant differences by one way or two way analysis of variance as appropriate followed by least significant difference tests on comparisons with the control and/or basal groups. *p* < 0.05 was the significance threshold. All analyses were carried out with GraphPad Prism 6 (San Diego, CA, USA).

## 3. Results

### 3.1. IC and SDS Induce Proinflammatory Cytokine Production in IEC18 Cells through a Pathway Involving the Activation of NF-κB

Culturing IEC18 cells, an intestinal epithelial cell line, with IC or SDS induced the production of the proinflammatory cytokines GROα and MCP-1 (Figure 1A,B, respectively). The magnitude of cytokine induction was similar for both products in the case of MCP-1, but SDS was a less powerful inducer of GROα than IC.

To characterize the connection of the key transcription factor NF-κB with the effect of IC and SDS on MCP-1, Bay-11-7082 (an inhibitor of IκB-α phosphorylation and of the ubiquitin system) was used. Our results indicate that NF-κB is clearly involved in the observed effects since its inhibition abrogated GROα and MCP-1 responses (Figure 1C,D, respectively).

MAPK and AKT are also involved in signal transduction to regulate the immune response to some extent, particularly in the case of MCP-1. Thus, inhibition of all three MAPK resulted in 50–70% inhibition of SDS-evoked MCP-1 secretion (significant only for ERK1/2), while it had no effect under IC stimulation. In turn, GROα was only modestly affected by the inhibition of JNK and/or ERK1/2. AKT inhibition potentiated IC-evoked MCP-1 secretion (Figure 1C,D).

#### 3.1.1. TLR4 Is Involved in the Induction of MCP-1 by SDS in IEC18 Cells

To characterize the contribution of TLRs to cytokine stimulation by IC or SDS we silenced the expression of TLR4 and MyD88, a canonical adaptor downstream of members of the TLR and IL-1 receptor families, in IEC18 cells. The impact on MCP-1 was assessed (selected on the basis of higher induction and similar regulation as GROα). Our results indicate a different mechanism of both products. Thus, silencing TLR4 significantly decreased IC-induced MCP-1 release. Nevertheless, a complete abrogation of the effect was not observed, pointing at a minor involvement of other pathways and/or receptors. In turn, no effect of TLR4 silencing on the response to SDS was observed, ruling out the contribution of this receptor. Silencing MyD88 almost entirely inhibited MCP-1 production evoked by either IC or SDS (Figure 1E), implying the involvement of other receptors that use MyD88 as an adaptor molecule.

#### 3.1.2. SDS and IC Regulate Cytokine Production in Spleen Cells

**Figure 2:**
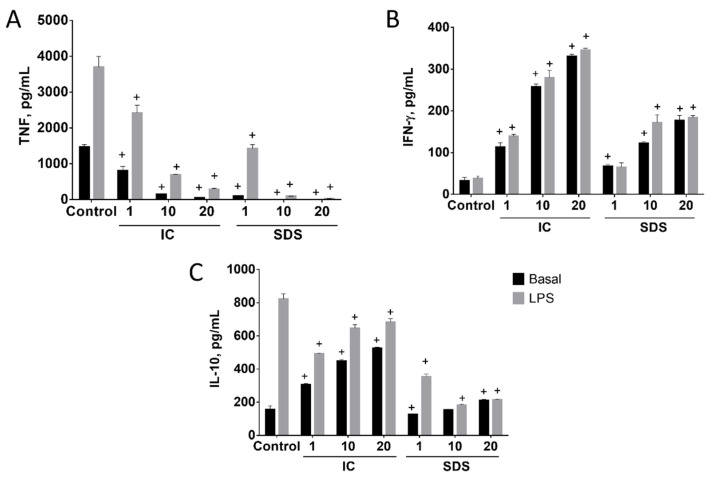
Effects of immunoglobulin concentrate (IC) and spray dried serum (SDS) on the production of TNF (**A**), IFN-γ (**B**), and IL-10 (**C**) in basal and LPS-stimulated rat splenocytes. IC and SDS (1, 10, and 20 mg/mL) were added to the cell culture medium and cytokines were measured in the supernatant after 24 h by ELISA. LPS (1 µg/mL) was added to stimulate cells 2 h after IC/SDS. Data are represented as mean ± SEM and are representative of two independent experiments (*n* = 6). ^+^ *p* < 0.05 vs. Control group.

The effects of IC and SDS on cytokine production by spleen cells depended on the product assayed and the cytokine measured (Figure 2). SDS and especially IC increased splenocyte IFN-γ production in basal conditions in all the concentrations assayed (Figure 2B). IC also induced IL-10 production, while only a small and inconsistent effect of SDS was observed (Figure 2C). In contrast, both IC and SDS were found to inhibit basal TNF production (Figure 2A). SDS was more potent than IC, as ~50% inhibition was achieved with a 10-fold lower concentration.

LPS induced the production of IL-10 and TNF, but not IFN-γ, by splenocytes (Figure 2). Different effects on IL-10 production were observed when LPS-treated cells were cultured in the presence of IC or SDS. Thus, SDS was found to inhibit LPS-evoked induction in a concentration-dependent fashion, while IC produced a small but significant decrease of IL-10 output (Figure 2C). In contrast, both products showed a similar effect profile regarding the production of TNF and IFN-γ in the presence or absence of LPS, clearly inhibiting the production of TNF while inducing IFN-γ (Figure 2). It is worth noting that TNF inhibition occurred despite the upregulation by LPS (Figure 2B).

**Figure 3:**
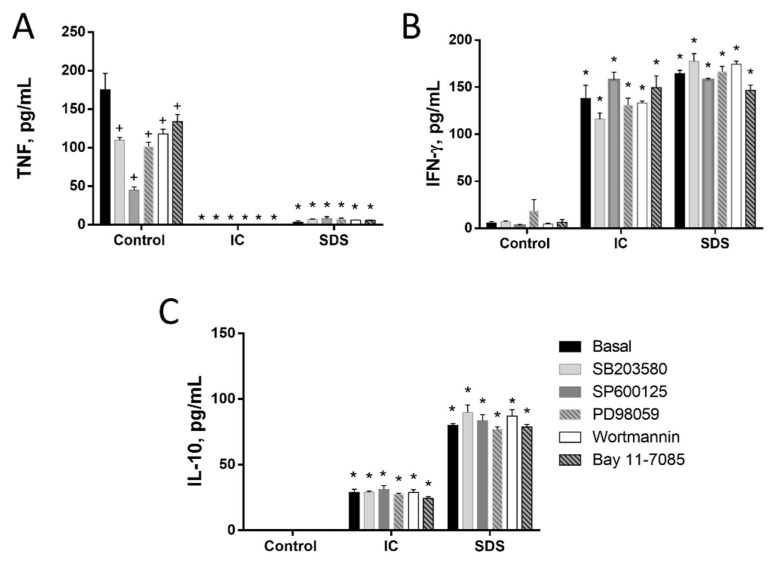
Effects of immunoglobulin concentrate (IC) and spray dried serum (SDS) on the production of TNF (**A**), IFN-γ (**B**), and IL-10 (**C**) by rat lymphocytes. IC and SDS (10 mg/mL) were added to the cell culture medium. Vehicle (DMSO) or inhibitors for p38 MAPK (SB203580, 10 µM), JNK (SP600125, 10 µM), ERK1/2 (PD98059, 10 µM), phosphatidylinositol 3-kinase (wortmannin, 1 μM) or NF-κB (Bay 11–7085, 10 μM) were added to rat lymphocytes 2 h before the addition of IC or SDS. Cytokines were measured by ELISA in the 24 h supernatant. Data are represented as mean ± SEM and are representative of two independent experiments (*n* = 3). * *p* < 0.05 vs. the corresponding Control group; ^+^ *p* < 0.05 vs. the corresponding Basal (no inhibitor) group.

**Figure 4:**
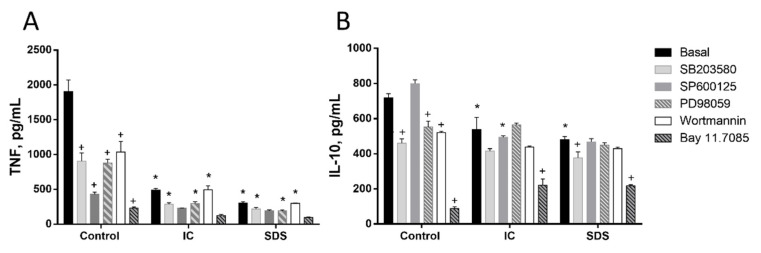
Effects of immunoglobulin concentrate (IC) and spray dried serum (SDS) on the production of TNF (**A**) and IL-10 (**B**) by rat monocytes. IC and SDS (10 mg/mL) were added to the cell culture medium and cytokines were measured in the supernatant after 24 h by ELISA. Vehicle (DMSO) or inhibitors for p38 MAPK (SB203580, 10 µM), JNK (SP600125, 10 µM), ERK1/2 (PD98059, 10 µM), phosphatidylinositol 3-kinase (wortmannin, 1 μM), or NF-κB (Bay 11–7085, 10 μM) were added to monocytes 2 h before the addition of IC or SDS. Data are represented as mean ± SEM and are representative of two independent experiments (*n* = 3). * *p* < 0.05 vs. the corresponding Control group; ^+^ *p* < 0.05 vs. the corresponding Basal (no inhibitor) group.

Splenocytes are a mix of several cell types. Among them T lymphocytes and monocytes are key cells of the immune adaptive and innate response, respectively. We isolated both cell types from the spleen and used these primary cultures to study the effect of IC and SDS on cytokine production. As in splenocytes, the addition of either IC or SDS to rat T lymphocytes enhanced IFN-γ and IL-10 production and decreased that of TNF (Figure 3). Monocytes were also isolated and cultured in similar conditions. In this case TNF and IL-10 production was downregulated in the presence of IC and SDS (Figure 4).

#### 3.1.3. Signal Transduction Pathways Involving NF-κB, MAPK, AKT, or RAGE Do Not Contribute to Cytokine Regulation by IC or SPD in Spleen Cells

**Figure 5:**
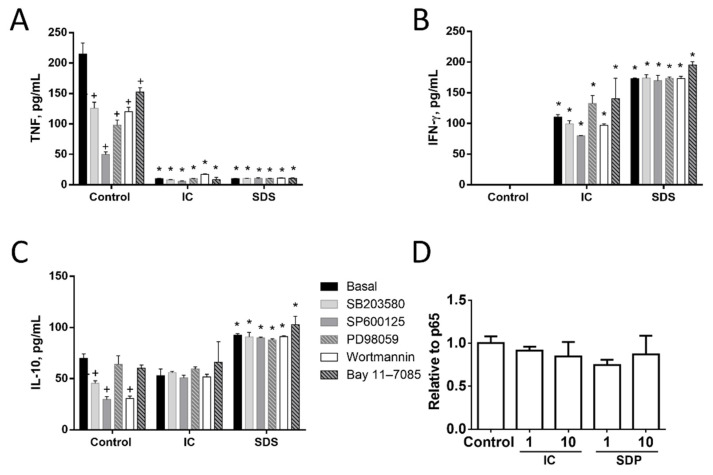
Effects of immunoglobulin concentrate (IC) and spray dried serum (SDS) on the production of TNF (**A**), IFN-γ (**B**), and IL-10 (**C**) by rat splenocytes. IC or SDS (10 mg/mL) were added to the cell culture medium. Vehicle (DMSO) or inhibitors for p38 MAPK (SB203580, 10 µM), JNK (SP600125, 10 µM), ERK1/2 (PD98059, 10 µM), phosphatidylinositol 3-kinase (wortmannin, 1 μM) or NF-κB (Bay 11–7085, 10 μM) were added to rat splenocytes 2 h before the addition of IC or SDS and cytokines were measured by ELISA in the 24 h supernatant. (**D**) Effect of IC and SDS on NF-κB p65 translocation to the nucleus. Splenocytes were incubated with IC and SDS for 15 minutes and nuclear content was extracted for p65 detection by ELISA. Data are represented as mean ± SEM and are representative of two independent experiments (*n* = 3). * *p* < 0.05 vs. the corresponding Control group; ^+^ *p* < 0.05 vs. the corresponding Basal (no inhibitor) group.

Next, we studied the molecular mechanisms involved in the observed responses using inhibitors of different signal transduction molecules involved in NF-κB, MAPK, AKT, or RAGE pathways (Figure 3, Figure 4 and Figure 5). As expected, the inhibitors downregulated basal cytokine production to various degrees. Nevertheless, inhibitors failed to counteract the effects of IC or SDS in splenocytes, T lymphocytes, or monocytes, indicating that these pathways are not involved in the effect. Since NF-κB is a key factor in the regulation of inflammation we further ruled out its involvement characterizing the effect of both products on p65 translocation to the nucleus, however, no induction was observed (Figure 5D).

#### 3.1.4. Induction of IL-10 Is Not Linked to TNF Inhibition by IC in Spleen Cells

**Figure 6:**
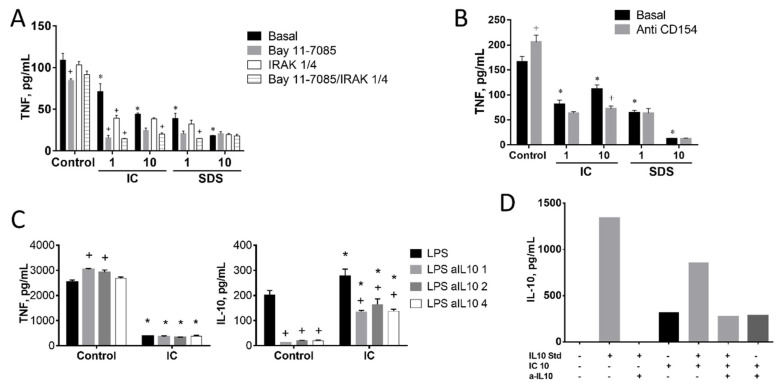
Involvement of IL-10 on the inhibition of TNF production by immunoglobulin concentrate (IC) and spray dried serum (SDS) in rat splenocytes. (**A**) Effect of NF-κB and IRAK1/4 inhibitors on TNF production. Bay 11-7082 (10 µM) and the IRAK 1/4 inhibitor 1 (10 µM) were added to the cell culture medium together with SDS and IC for 48 h. (**B**) Effect of a blocking anti-CD154 antibody on TNF production, following the same protocol. (**C**) Effect of a blocking anti-IL-10 antibody (1, 2 or 4 µg/mL) on TNF and IL-10 secretion, following the same protocol. (**D**) Evaluation of IC immunoreactivity by rat IL-10 ELISA and blockade by rat anti-IL-10 antibody. IC (10 mg/mL) was measured with a rat ELISA as described in Materials and Methods and the signal compared with that of the rat IL-10 ELISA standard (1250 pg/mL). The effect of incubation for 4 h at 37 °C with a blocking anti-IL10 antibody (1 µg/mL) was also assessed. RPMI medium without FBS was used in all cases. Cytokines were measured in the culture medium by ELISA. ^+^ *p* < 0.05 vs. the corresponding Basal group; * *p* < 0.05 vs. Control group.

It has been described that the stimulation of CD40 by T lymphocyte membrane-bound CD40L may induce IRAK1 and subsequently the production of IL-10. In turn, IL-10 may inhibit TNF expression by inducing the degradation of its mRNA [34]. To test the involvement of this pathway we cultured splenocytes in the presence of IC or SDS plus an inhibitor of IRAK1/4 (Figure 6A) or the CD40L-blocking antibody (Figure 6B). Neither of these molecules restored the production of TNF. In addition, the impact of an IL-10 blocking antibody on IC-induced TNF modulation was tested using three different concentrations of the former (1, 2, or 4 µg/mL, Figure 6C, left). An almost complete IL-10 blockade was achieved in all cases in the absence of IC (Figure 6C, right), resulting in a slight upregulation of TNF release, indicating a significant (albeit minor) modulation under LPS stimulation in splenocytes. Addition of IC resulted in the expected inhibition of TNF, which was associated with a slight enhancement of IL-10 production. However, IL-10 blockade had no effect on TNF downregulation by IC. Of note, IL-10 levels were higher in the medium of IC-treated cells.

One possible explanation for these results is that IC contains bioactive IL-10 of porcine origin. To explore this possibility IL-10 immunoreactivity was measured in IC with a rat IL-10 ELISA (Figure 6D). A positive signal was detected, which was not inhibited by the anti-IL-10 antibody, unlike the rat IL-10 standard. This suggests that IC does contain porcine IL-10, but this needs confirmation and quantification by specific antibody (not available).

## 4. Discussion

Using IEC18 rat ileal cells as model intestinal epithelial cells we have described that both protein concentrates upregulate GROα and MCP-1 chemokine production in basal conditions, an effect that is mediated by NF-κB and requires MyD88. MyD88 is an adaptor molecule for innate immune IL-1 receptors and all TLRs except TLR3, linking these receptors to IRAK, that in turn activates NF-κB and MAPK. In turn, TLR4 was required for IC but not SDS effects. In this regard our results indicate that IC/SDS may similarly elicit GROα and MCP-1 secretion acting from the luminal side in vivo. This is contingent on these products reaching the intestine in sufficiently high concentrations. ICs have been demonstrated to be digested only partially and to reach the distal sites of the gastrointestinal tract [26,27,28]. The immunomodulatory activity of other proteins has also been shown to be partly resistant to digestion [32,35]. Are the concentrations tested in our study relevant in vivo? Quantitatively speaking, a supplementation of 80 g/kg and 22.7 mg/kg diet for SDS and IC as in the in vivo study of Pérez-Bosque et al. [9] and others would result in 240 and 68.1 mg/day at the standard intake of 3 g/d in mice. If no degradation was produced, this would be expected to produce concentrations of approximately 1200 and 340.5 mg/mL in the colon (assuming a volume of 0.3 mL). In other words, only around 3% of the intake of IC is required to yield concentrations shown to be active in the present in vitro study. Interestingly, porcine SDS has been recently shown in a multi-OMICS study to modulate skin barrier function in teleost fish when given as a nutritional supplement [36]. This study not only supports the bioactivity of the components of this product beyond the epithelial layer, but shows an effect comparable to that in the intestinal mucosa, which shares a number of characteristics with the skin as a biological barrier.

Stimulation of MyD88-related receptors and NF-κB in enterocytes by different molecules serves a series of complex immunomodulatory and physiological regulatory purposes, including not only cytokine/chemokine secretion, but also the regulation of apoptosis and proliferation in IECs [37] and antibacterial peptide secretion [38,39,40]. Thus it is likely that other MyD88/NFκB-dependent beneficial responses may be at play in IC/SDS protective effects at the epithelial level, although this will require additional experiments. Thus the intestinal epithelial effects of SDS and IC are consistent with reinforcement of mucosal barrier function and overall protection against inflammation [41,42]. This effect resembles microbiota-derived signals that, sensed to a great extent via TLRs, contribute significantly to the regulation of epithelial cell turnover and resistance to mucosal injury [43]. Importantly, TLR4 KO mice exhibit increased sensitivity to experimental colitis, along with various other TLRs and related receptors and signaling molecules [42]. Similarly, a number of compounds with functional foods properties have been described to act as TLR4 ligands or activators having no deleterious effect in vivo (such as colitis) while limiting experimental intestinal inflammation, including bovine glycomacropeptide, nonabsorbable carbohydrates or active hexose correlated compound [33,44,45,46,47,48,49,50,51,52]. As indicated, this is precisely the case also for these porcine protein concentrates in vivo [2,8,9].

On the other hand, SDS and IC were found to consistently inhibit TNF production by rat splenocytes, T lymphocytes, and monocytes. This effect was associated with increased IL-10 and IFN-γ production in splenocytes and T lymphocytes, while IL-10 was inhibited in monocytes. TNF is a target for anti-inflammatory therapies, particularly anti-TNF antibodies. Because TNF inhibition effects were so consistent, we aimed to better characterize the molecular pathways involved, finding that NF-κB, MAPKs, RAGE, or AKT were not involved. This was also the case for IFN-γ production. It has been described that T cells down-regulate monocyte TNF production by IRAK1-mediated IL-10 expression [53]. TNF decrease was however shown to be totally independent of IRAK4 inhibition and also to be not reversed by CD154/CD40L blockade (which actually enhanced TNF lowering by IC). Perhaps more importantly, the effect on TNF was unchanged by addition of a blocking antibody against IL-10 itself (the latter documented only for IC). It is noteworthy that in this experiment while the anti-IL-10 antibody virtually eliminated IL-10 from the culture medium in the control (i.e., LPS only) group, it did so only by ~50% in the IC groups, despite the fact that IL-10 levels were quite similar in both. One possible explanation is that IC may contain biologically active porcine IL-10, which may be unaffected by the anti-IL-10 antibody but detected by the rat IL-10 ELISA. Such cross-species sensitivity is not contemplated in either the ELISA assay or the anti-IL10 antibody. However, at 10 g/L IC does show a positive signal in the rat IL-10 ELISA, which is not affected by the anti-IL10 antibody. Thus this is a possible mechanism. Since TNF is a pivotal proinflammatory cytokine in intestinal inflammation, it is possible that SDS/IC may act in part by downregulating this key player in the inflammatory response. This would obviously require access of these products to the mucosal milieu. This remains uncertain at this point, but it is a mechanism probably facilitated by the disruption of the mucosal barrier in an inflammatory context.

It is interesting to comment the similar composition of porcine concentrates and that of intravenous immunoglobulin treatment (IVIg) therapy. This therapy consists in the administration of human immunoglobulins (mainly IgG) and has proven effective in the treatment of a wide variety of autoimmune and chronic inflammatory diseases. Unfortunately, the mechanism of action of IVIg remains poorly characterized, although it is thought to involve a complex interplay with the host immune system [54]. Of note, a similar approach has been aplied with animal protein concentrates by the oral route. Recently this strategy has been tried in noncritical COVID19 patients (ClinicalTrials.gov Identifier: NCT04682041, accessed on 4 March 2021).

Our research group has previously shown that several protein products can directly regulate the immune response of intestinal epithelial cells, monocytes, and lymphocytes. Among these products are protein fractions, a protein hydrolysate from algae, and bovine glycomacropeptide, a 64 amino acid peptide that is part of κ-casein as obtained in whey in the cheese-making process [33,46,49,50,55,56,57]. These products induce the production of IL-10 by monocytes/macrophages and lymphocytes by way of NF-κB and MAPK (p38 and JNK) activation [32,50,55]. At the same time, they inhibit the production of proinflammatory cytokines by monocytes/macrophages. Thus, there is overlap between the effects of these protein products in vitro.

Our data provide new evidence about the possible mechanism whereby SDS and IC may protect against colitis and reinforce barrier function. As discussed, the efficacy of porcine protein concentrates when administered by the oral route in vivo depends on the capacity to withstand digestion and, for nonepithelial actions, to access the intestinal mucosa. Protein concentrates may have additional mechanisms, such as modulation of the microbiota, adsorption/neutralization of toxins, etc. In fact, prebiotic effects have been documented in the case of porcine SDP [58].

## 5. Conclusions

In conclusion, our results indicate that our hypothesis, i.e., dietary plasma protein concentrates from animal origin may have immunomodulatory activity, is correct. Serum protein concentrates were shown to exert complex biological immunomodulatory actions in vitro. One is represented by the activation of MyD88 and NFκB in IECs. This effect may contribute to the reinforcement of the intestinal barrier function. The actions on splenocytes (inducing IL-10 and inhibiting TNF) may result in anti-inflammatory effects.

## Data Availability

Data is contained within the article.

## Abbreviations

DMEM: Dulbecco’s modified Eagle’s medium; DMSO, dimethyl sulfoxide; ELISA, enzyme-linked immunoassay; GROα, growth regulated oncogene α; IC, immunoglobulin concentrate; IL, interleukin; LPS, lipopolysaccharide; MAPK, mitogen activated protein kinase; MCP-1, monocyte chemoattractant protein 1; PI3K, phosphatidylinositol 3 kinase; RAGE, advanced glycation end products; RPMI, Roswell Park Memorial Institute; SDP, spray dried plasma; SDS, spray dried serum.

## References

[1] Corl B.A., Harrell R.J., Moon H.K., Phillips O., Weaver E.M., Campbell J.M., Arthington J.D., Odle J. (2007). Effect of animal plasma proteins on intestinal damage and recovery of neonatal pigs infected with rotavirus. J. Nutr. Biochem..

[2] Perez-Bosque A., Miro L., Polo J., Russell L., Campbell J., Weaver E., Crenshaw J., Moreto M. (2010). Dietary plasma protein supplements prevent the release of mucosal proinflammatory mediators in intestinal inflammation in rats. J. Nutr..

[3] Perez-Bosque A., Moreto M. (2010). A rat model of mild intestinal inflammation induced by *Staphylococcus aureus* enterotoxin B. Proc. Nutr. Soc..

[4] Maijo M., Miro L., Polo J., Campbell J., Russell L., Crenshaw J., Weaver E., Moreto M., Perez-Bosque A. (2012). Dietary plasma proteins attenuate the innate immunity response in a mouse model of acute lung injury. Br. J. Nutr..

[5] Maijo M., Miro L., Polo J., Campbell J., Russell L., Crenshaw J., Weaver E., Moreto M., Perez-Bosque A. (2012). Dietary plasma proteins modulate the adaptive immune response in mice with acute lung inflammation. J. Nutr..

[6] Moreto M., Perez-Bosque A. (2009). Dietary plasma proteins, the intestinal immune system, and the barrier functions of the intestinal mucosa. J. Anim. Sci..

[7] Perez-Bosque A., Miro L., Amat C., Polo J., Moreto M. (2016). The Anti-Inflammatory Effect of Spray-Dried Plasma Is Mediated by a Reduction in Mucosal Lymphocyte Activation and Infiltration in a Mouse Model of Intestinal Inflammation. Nutrients.

[8] Miro L., Garcia-Just A., Amat C., Polo J., Moreto M., Perez-Bosque A. (2017). Dietary Animal Plasma Proteins Improve the Intestinal Immune Response in Senescent Mice. Nutrients.

[9] Perez-Bosque A., Amat C., Polo J., Campbell J.M., Crenshaw J., Russell L., Moreto M. (2006). Spray-dried animal plasma prevents the effects of Staphylococcus aureus enterotoxin B on intestinal barrier function in weaned rats. J. Nutr..

[10] Perez-Bosque A., Miro L., Maijo M., Polo J., Campbell J., Russell L., Crenshaw J., Weaver E., Moreto M. (2015). Dietary intervention with serum-derived bovine immunoglobulins protects barrier function in a mouse model of colitis. Am. J. Physiol. Gastrointest. Liver Physiol..

[11] Perez-Bosque A., Miro L., Maijo M., Polo J., Campbell J.M., Russell L., Crenshaw J.D., Weaver E., Moreto M. (2016). Oral Serum-Derived Bovine Immunoglobulin/Protein Isolate Has Immunomodulatory Effects on the Colon of Mice that Spontaneously Develop Colitis. PLoS ONE.

[12] Miró L., Amat C., Rosell-Cardona C., Campbell J.M., Polo J., Pérez-Bosque A., Moretó M. (2020). Dietary Supplementation with Spray-Dried Porcine Plasma Attenuates Colon Inflammation in a Genetic Mouse Model of Inflammatory Bowel Disease. Int. J. Mol. Sci..

[13] Bateman E., Weaver E., Klein G., Wignall A., Wozniak B., Plews E., Mayo B., White I., Keefe D. (2016). Serum-derived bovine immunoglobulin/protein isolate in the alleviation of chemotherapy-induced mucositis. Support. Care Cancer Off. J. Multinatl. Assoc. Support. Care Cancer.

[14] Miró L., Amat C., Polo J., Moretó M., Pérez-Bosque A. (2020). Anti-inflammatory effects of animal plasma protein supplementation in mice undergoing simultaneous gut and lung inflammation. Food Agric. Immunol..

[15] Garcia-Just A., Miró L., Pérez-Bosque A., Amat C., Polo J., Pallàs M., Griñán-Ferré C., Moretó M. (2020). Dietary Spray-Dried Porcine Plasma Prevents Cognitive Decline in Senescent Mice and Reduces Neuroinflammation and Oxidative Stress. J. Nutr..

[16] Liu Y., Choe J., Lee J.J., Kim J., Campbell J.M., Polo J., Crenshaw J.D., Pettigrew J.E., Song M. (2018). Spray-dried plasma attenuates inflammation and lethargic behaviors of pregnant mice caused by lipopolysaccharide. PLoS ONE.

[17] Jung T.H., Choi J.H., Koh K.C., Jeon W.M., Han K.S. (2017). Purification and Anti-pathogenic Properties of Immunoglobulin Concentrates from Porcine Blood. Korean J. Food Sci. Anim. Resour..

[18] Han K.S., Boland M., Singh H., Moughan P.J. (2009). The in vitro anti-pathogenic activity of immunoglobulin concentrates extracted from ovine blood. Appl. Biochem. Biotechnol..

[19] Quigley J.D., Strohbehn R.E., Kost C.J., O’Brien M.M. (2001). Formulation of colostrum supplements, colostrum replacers and acquisition of passive immunity in neonatal calves. J. Dairy Sci..

[20] Good L., Rosario R., Panas R. (2015). New therapeutic option for irritable bowel syndrome: Serum-derived bovine immunoglobulin. World J. Gastroenterol..

[21] Petschow B.W., Burnett B.P., Shaw A.L., Weaver E.M., Klein G.L. (2015). Dietary requirement for serum-derived bovine immunoglobulins in the clinical management of patients with enteropathy. Dig. Dis. Sci..

[22] Greenberg P.D., Cello J.P. (1996). Treatment of severe diarrhea caused by Cryptosporidium parvum with oral bovine immunoglobulin concentrate in patients with AIDS. J. Acquir. Immune. Defic. Syndr. Hum. Retrovirol..

[23] Tacket C.O., Binion S.B., Bostwick E., Losonsky G., Roy M.J., Edelman R. (1992). Efficacy of bovine milk immunoglobulin concentrate in preventing illness after Shigella flexneri challenge. Am. J. Trop Med. Hyg..

[24] Tacket C.O., Losonsky G., Link H., Hoang Y., Guesry P., Hilpert H., Levine M.M. (1988). Protection by milk immunoglobulin concentrate against oral challenge with enterotoxigenic *Escherichia coli*. N. Engl. J. Med..

[25] Tawfeek H.I., Najim N.H., Al-Mashikhi S. (2003). Efficacy of an infant formula containing anti-*Escherichia coli* colostral antibodies from hyperimmunized cows in preventing diarrhea in infants and children: A field trial. Int. J. Infect. Dis..

[26] Roos N., Mahé S., Benamouzig R., Sick H., Rautureau J., Tomé D. (1995). 15N-labeled immunoglobulins from bovine colostrum are partially resistant to digestion in human intestine. J. Nutr..

[27] Warny M., Fatimi A., Bostwick E.F., Laine D.C., Lebel F., LaMont J.T., Pothoulakis C., Kelly C.P. (1999). Bovine immunoglobulin concentrate-clostridium difficile retains C difficile toxin neutralising activity after passage through the human stomach and small intestine. Gut.

[28] Kelly C.P., Chetham S., Keates S., Bostwick E.F., Roush A.M., Castagliuolo I., LaMont J.T., Pothoulakis C. (1997). Survival of anti-Clostridium difficile bovine immunoglobulin concentrate in the human gastrointestinal tract. Antimicrob. Agents Chemother..

[29] Henderson A.L., Brand M.W., Darling R.J., Maas K.J., Detzel C.J., Hostetter J., Wannemuehler M.J., Weaver E.M. (2015). Attenuation of Colitis by Serum-Derived Bovine Immunoglobulin/Protein Isolate in a Defined Microbiota Mouse Model. Dig. Dis. Sci..

[30] Ustunol Z., Wong C. (2010). Effect of nonfat dry milk and major whey components on interleukin-6 and interleukin-8 production in human intestinal epithelial-like Caco-2 cells. J. Dairy Sci..

[31] Lee Y.Z., Sim J.S., Al-Mashikhi S., Nakai S. (1988). Separation of immunoglobulins from bovine blood by polyphosphate precipitation and chromatography. J. Agric. Food Chem..

[32] Cian R.E., Hernandez-Chirlaque C., Gamez-Belmonte R., Drago S.R., Sanchez de Medina F., Martinez-Augustin O. (2018). Green Alga Ulva spp. Hydrolysates and Their Peptide Fractions Regulate Cytokine Production in Splenic Macrophages and Lymphocytes Involving the TLR4-NFkappaB/MAPK Pathways. Mar. Drugs.

[33] Daddaoua A., Martinez-Plata E., Ortega-Gonzalez M., Ocon B., Aranda C.J., Zarzuelo A., Suarez M.D., de Medina F.S., Martinez-Augustin O. (2013). The nutritional supplement Active Hexose Correlated Compound (AHCC) has direct immunomodulatory actions on intestinal epithelial cells and macrophages involving TLR/MyD88 and NF-kappaB/MAPK activation. Food Chem..

[34] Foey A.D., Feldmann M., Brennan F.M. (2001). CD40 ligation induces macrophage IL-10 and TNF-alpha production: Differential use of the PI3K and p42/44 MAPK-pathways. Cytokine.

[35] Cian R.E., Garzón A.G., Martínez-Augustin O., Botto C.C., Drago S.R. (2018). Antithrombotic Activity of Brewers’ Spent Grain Peptides and their Effects on Blood Coagulation Pathways. Plant. Foods Hum. Nutr..

[36] Reyes-López F.E., Ibarz A., Ordóñez-Grande B., Vallejos-Vidal E., Andree K.B., Balasch J.C., Fernández-Alacid L., Sanahuja I., Sánchez-Nuño S., Firmino J.P. (2020). Skin Multi-Omics-Based Interactome Analysis: Integrating the Tissue and Mucus Exuded Layer for a Comprehensive Understanding of the Teleost Mucosa Functionality as Model of Study. Front. Immunol..

[37] Günther C., Buchen B., Neurath M.F., Becker C. (2014). Regulation and pathophysiological role of epithelial turnover in the gut. Semin. Cell Dev. Biol..

[38] Menendez A., Willing B.P., Montero M., Wlodarska M., So C.C., Bhinder G., Vallance B.A., Finlay B.B. (2013). Bacterial stimulation of the TLR-MyD88 pathway modulates the homeostatic expression of ileal Paneth cell alpha-defensins. J. Innate Immun..

[39] Omagari D., Takenouchi-Ohkubo N., Endo S., Ishigami T., Sawada A., Moro I., Asano M., Komiyama K. (2011). Nuclear factor kappa B plays a pivotal role in polyinosinic-polycytidylic acid-induced expression of human beta-defensin 2 in intestinal epithelial cells. Clin. Exp. Immunol..

[40] Gong J., Xu J., Zhu W., Gao X., Li N., Li J. (2010). Epithelial-specific blockade of MyD88-dependent pathway causes spontaneous small intestinal inflammation. Clin. Immunol..

[41] Martinez-Augustin O., Rivero-Gutierrez B., Mascaraque C., Sanchez de Medina F. (2014). Food derived bioactive peptides and intestinal barrier function. Int. J. Mol. Sci..

[42] Sanchez de Medina F., Romero-Calvo I., Mascaraque C., Martinez-Augustin O. (2014). Intestinal inflammation and mucosal barrier function. Inflamm. Bowel. Dis..

[43] Hernandez-Chirlaque C., Aranda C.J., Ocon B., Capitan-Canadas F., Ortega-Gonzalez M., Carrero J.J., Suarez M.D., Zarzuelo A., Sanchez de Medina F., Martinez-Augustin O. (2016). Germ-free and Antibiotic-treated Mice are Highly Susceptible to Epithelial Injury in DSS Colitis. J. Crohn’s Colitis.

[44] Capitan-Canadas F., Ocon B., Aranda C.J., Anzola A., Suarez M.D., Zarzuelo A., de Medina F.S., Martinez-Augustin O. (2016). Fructooligosaccharides exert intestinal anti-inflammatory activity in the CD4+ CD62L+ T cell transfer model of colitis in C57BL/6J mice. Eur. J. Nutr..

[45] Ortega-Gonzalez M., Capitan-Canadas F., Requena P., Ocon B., Romero-Calvo I., Aranda C., Suarez M.D., Zarzuelo A., Sanchez de Medina F., Martinez-Augustin O. (2014). Validation of bovine glycomacropeptide as an intestinal anti-inflammatory nutraceutical in the lymphocyte-transfer model of colitis. Br. J. Nutr..

[46] Ortega-Gonzalez M., Ocon B., Romero-Calvo I., Anzola A., Guadix E., Zarzuelo A., Suarez M.D., Sanchez de Medina F., Martinez-Augustin O. (2014). Nondigestible oligosaccharides exert nonprebiotic effects on intestinal epithelial cells enhancing the immune response via activation of TLR4-NFkappaB. Mol. Nutr. Food Res..

[47] Mascaraque C., Aranda C., Ocon B., Monte M.J., Suarez M.D., Zarzuelo A., Marin J.J., Martinez-Augustin O., Sanchez de Medina F. (2014). Rutin has intestinal antiinflammatory effects in the CD4+ CD62L+ T cell transfer model of colitis. Pharm. Res..

[48] Mascaraque C., Suarez M.D., Zarzuelo A., Sanchez de Medina F., Martinez-Augustin O. (2014). Active hexose correlated compound exerts therapeutic effects in lymphocyte driven colitis. Mol. Nutr. Food Res..

[49] Lopez-Posadas R., Requena P., Gonzalez R., Suarez M.D., Zarzuelo A., Sanchez de Medina F., Martinez-Augustin O. (2010). Bovine glycomacropeptide has intestinal antiinflammatory effects in rats with dextran sulfate-induced colitis. J. Nutr..

[50] Requena P., Daddaoua A., Guadix E., Zarzuelo A., Suarez M.D., Sanchez de Medina F., Martinez-Augustin O. (2009). Bovine glycomacropeptide induces cytokine production in human monocytes through the stimulation of the MAPK and the NF-kappaB signal transduction pathways. Br. J. Pharm..

[51] Requena P., Daddaoua A., Martínez-Plata E., González M., Zarzuelo A., Suárez M.D., Sánchez de Medina F., Martínez-Augustin O. (2008). Bovine glycomacropeptide ameliorates experimental rat ileitis by mechanisms involving downregulation of interleukin 17. Br. J. Pharmacol..

[52] Daddaoua A., Puerta V., Zarzuelo A., Suarez M.D., Sanchez de Medina F., Martinez-Augustin O. (2005). Bovine glycomacropeptide is anti-inflammatory in rats with hapten-induced colitis. J. Nutr..

[53] Inoue M., Arikawa T., Chen Y.H., Moriwaki Y., Price M., Brown M., Perfect J.R., Shinohara M.L. (2014). T cells down-regulate macrophage TNF production by IRAK1-mediated IL-10 expression and control innate hyperinflammation. Proc. Natl. Acad. Sci. USA.

[54] Bayry J., Thirion M., Misra N., Thorenoor N., Delignat S., Lacroix-Desmazes S., Bellon B., Kaveri S., D Kazatchkine M. (2003). Mechanisms of Action of Intravenous Immunoglobulin in Autoimmune and Inflammatory Diseases. Transfus. Clin. Biol..

[55] Requena P., Gonzalez R., Lopez-Posadas R., Abadia-Molina A., Suarez M.D., Zarzuelo A., de Medina F.S., Martinez-Augustin O. (2010). The intestinal antiinflammatory agent glycomacropeptide has immunomodulatory actions on rat splenocytes. Biochem. Pharm..

[56] Cian R.E., Lopez-Posadas R., Drago S.R., Medina F.S., Martinez-Augustin O. (2012). Immunomodulatory Properties of the Protein Fraction from Phorphyra columbina. J. Agric. Food Chem..

[57] Cian R.E., Lopez-Posadas R., Drago S.R., Sanchez de Medina F., Martinez-Augustin O. (2012). A Porphyra columbina hydrolysate upregulates IL-10 production in rat macrophages and lymphocytes through an NF-kappaB, and p38 and JNK dependent mechanism. Food Chem..

[58] Moretó M., Miró L., Amat C., Polo J., Manichanh C., Pérez-Bosque A. (2020). Dietary supplementation with spray-dried porcine plasma has prebiotic effects on gut microbiota in mice. Sci. Rep..

